# Genetic insights of H9N2 avian influenza viruses circulating in Mali and phylogeographic patterns in Northern and Western Africa

**DOI:** 10.1093/ve/veae011

**Published:** 2024-02-19

**Authors:** Idrissa Nonmon Sanogo, Claire Guinat, Simon Dellicour, Mohamed Adama Diakité, Mamadou Niang, Ousmane A Koita, Christelle Camus, Mariette Ducatez

**Affiliations:** Interactions Hôtes-Agents Pathogènes (IHAP), UMR 1225, Université de Toulouse, INRAE, ENVT, Toulouse 31076, France; Faculté d’Agronomie et de Médecine Animale (FAMA), Université de Ségou, Ségou BP 24, Mali; Interactions Hôtes-Agents Pathogènes (IHAP), UMR 1225, Université de Toulouse, INRAE, ENVT, Toulouse 31076, France; Spatial Epidemiology Lab (SpELL), Université Libre de Bruxelles, Brussels B-1050, Belgium; Department of Microbiology, Immunology and Transplantation, Rega Institute, Laboratory for Clinical and Epidemiological Virology, KU Leuven, Leuven BE-3000, Belgium; Service diagnostic et recherche Laboratoire Central Vétérinaire, Bamako BP 2295, Mali; Food and Agriculture Organization of the United Nations (FAO-UN), Emergency Centre for Transboundary Animal Diseases (ECTAD), Regional Office for Africa (RAF), Accra BP 1628, Ghana; Laboratoire de Biologie Moléculaire Appliquée, Faculté des Sciences et Techniques (FAST), University of Sciences, Techniques and Technologies of Bamako (USTTB), Mali Université de Bamako, Bamako E 3206, Mali; Interactions Hôtes-Agents Pathogènes (IHAP), UMR 1225, Université de Toulouse, INRAE, ENVT, Toulouse 31076, France; Interactions Hôtes-Agents Pathogènes (IHAP), UMR 1225, Université de Toulouse, INRAE, ENVT, Toulouse 31076, France

**Keywords:** influenza A virus, H9N2, molecular epidemiology, viral phylogeography, mali, western Africa

## Abstract

Avian influenza viruses (AIVs) of the H9N2 subtype have become widespread in Western Africa since their first detection in 2017 in Burkina Faso. However, the genetic characteristics and diffusion patterns of the H9N2 virus remain poorly understood in Western Africa, mainly due to limited surveillance activities. In addition, Mali, a country considered to play an important role in the epidemiology of AIVs in the region, lacks more comprehensive data on the genetic characteristics of these viruses, especially the H9N2 subtype. To better understand the genetic characteristics and spatio-temporal dynamics of H9N2 virus within this region, we carried out a comprehensive genetic characterization of H9N2 viruses collected through active surveillance in live bird markets in Mali between 2021 and 2022. We also performed a continuous phylogeographic analysis to unravel the dispersal history of H9N2 lineages between Northern and Western Africa. The identified Malian H9N2 virus belonged to the G1 lineage, similar to viruses circulating in both Western and Northern Africa, and possessed multiple molecular markers associated with an increased potential for zoonotic transmission and virulence. Notably, some Malian strains carried the R-S-N-R motif at their cleavage site, mainly observed in H9N2 strains in Asia. Our continuous phylogeographic analysis revealed a single and significant long-distance lineage dispersal event of the H9N2 virus to Western Africa, likely to have originated from Morocco in 2015, shaping the westward diffusion of the H9N2 virus. Our study highlights the need for long-term surveillance of H9N2 viruses in poultry populations in Western Africa, which is crucial for a better understanding of virus evolution and effective management against potential zoonotic AIV strain emergence.

## Introduction

Low pathogenic AIVs (LPAIVs), particularly the H9N2 subtype, pose a significant global threat to both poultry and human health (Pusch and Suarez 2018; Peacock et al. 2019). H9N2 viruses have the potential to cross species barriers and infect various mammalian species, including humans (Peiris et al. 1999; Qian et al. 2021). While most H9N2 viruses circulate asymptomatically in poultry and wild birds (Peacock et al. 2019), certain H9N2 strains have been associated with outbreaks causing high mortality among domestic poultry (El Houadfi et al. 2016; Perez and Wit de 2016; Jeevan et al. 2019).

In Africa, H9N2 virus was first reported in Egypt and Tunisia in 2011, marking the beginning of its detection in Northern Africa (Tombari et al. 2011; El-Zoghby et al. 2012; Monne et al. 2013). Subsequently, since its emergence in Morocco in 2016, the virus has rapidly disseminated across the country and to other regions in Africa (El Houadfi et al. 2016; Nagy, Mettenleiter, and Abdelwhab 2017). In Western Africa, the virus was initially detected in 2017 in Burkina Faso (Zecchin et al. 2017), followed by subsequent identification in Senegal (Jallow et al. 2020), Ghana (Awuni et al. 2019; Kotey et al. 2022), Nigeria (Sulaiman et al. 2021), Benin, and Togo (Fusade-Boyer et al. 2021). Notably, H9N2 viruses isolated in Western Africa so far belong to the G1 lineage and share genetic relatedness with those circulating in Northern Africa and the Middle East (El Houadfi et al. 2016; Barberis et al. 2020; El Mellouli et al. 2022b). However, the extent and mechanisms of geographical diffusion between Northern and Western Africa remain poorly understood, primarily due to limited surveillance activities in Western Africa, leading to under-reporting and underdetection of H9N2 viruses (Pavade et al. 2011).

Phylogeographic analyses of avian influenza virus (AIV) genome sequences have proven to be powerful tools for unveiling the complex spatio-temporal dynamics of these viruses. Specifically, phylogeography contributed to unraveling the origin, migration patterns, and dispersal history of H9N2 viruses in different regions such as Asia, the Middle East, and North Africa (Fusaro et al. 2011; Jin et al. 2014; Li et al. 2020; El Mellouli et al. 2022b). However, the application of these methodologies to understand the transmission dynamics and introduction drivers of the H9N2 viruses in Western Africa remains largely unexplored. Therefore, harnessing these analytical tools and data becomes essential to shed light on the dissemination dynamics of H9N2 viruses within the unique context of Western Africa (Yang et al. 2019).

Despite experiencing the circulation of LPAIVs in domestic poultry since 2007 (Molia et al. 2010), Mali lacks comprehensive studies on the epidemiology and genetic characteristics of these viruses, particularly H9N2. Indeed, previous studies related to AIVs in domestic and wild birds have mainly focused on the epidemiology of highly pathogenic avian influenza viruses (HPAIVs) and rarely addressed low pathogenic avian influenza viruses (LPAIVs). In addition, the country faces a heightened risk of new AIV strain emergence due to the predominant practice of rearing domestic birds in traditional farming systems without adequate biosecurity measures (Traoré 2013; Molia et al. 2015). This is coupled with the presence of the Inner Delta of the Niger River, which serves as a significant gathering site for millions of wild birds that may carry AIVs (Gaidet et al. 2007; Cappelle et al. 2012).

Live bird markets (LBMs) in Mali play a substantial role in the emergence and spread of new AIV strains, as various species of domestic birds from different regions are brought together (Molia et al. 2016). Risk factors associated with the presence of AIVs, such as poor sanitary conditions and improper disposal of dead birds, have been reported in the majority of Malian LBMs (Molia et al. 2016). Therefore, active surveillance in LBMs is crucial for understanding the genetic characteristics, geographic origins, and dispersal patterns of the circulating H9N2 viruses in Mali.

In this study, we conducted a comprehensive genomic characterization of H9N2 viruses collected from LBMs in Mali in 2022. Additionally, we performed a continuous phylogeographic analysis to unravel the dispersal history of H9N2 lineages between Northern and Western Africa. Our results indicate that the H9N2 virus detected in Mali belongs to the G1 lineage and shows a potential for zoonotic transmission. Additionally, we demonstrated that a single significant long-distance lineage dispersal event of the H9N2 virus to Western Africa originated from Morocco, shaping the westward diffusion of the H9N2 virus.

## Materials and methods

### H9N2 genome sequences from Mali

From June 2021 to April 2022, a total of 519 cloacal swabs were collected from apparently healthy chickens in eleven LBMs in Bamako, Ségou, and Mopti in Mali. Samples were usually collected on the same day each month from the same stall. The swabs were placed into sterile tubes containing 500 µl of phosphate-buffered saline supplemented with antibiotics (100 IU/ml penicillin: and streptomycin: 100 µg/ml). Samples were transported within 24 h to the Central Veterinary Laboratory (LCV) of Bamako and stored at −80°C until further analysis.

Viral RNA was extracted from the swabs using the NucleoSpin RNA extraction kit (Macherey-Nagel, Germany) according to the manufacturer’s instructions. The extracted RNA samples were tested in pools of five using real-time reverse-transcription polymerase chain reaction (RT-PCR) targeting the influenza A matrix (*M*) gene at the LCV (Fouchier et al. 2000). A total of 100 µl of positive samples were applied to Flinders Technology Associates (FTA) cards to inactivate viral infectivity and preserve nucleic acid integrity. The FTA cards were shipped to the VIRéMIE laboratory of UMR IHAP in Toulouse for further analysis. RNA was eluted from FTA cards by placing 6-mm-diameter disk fragments, cut from the FTA card spots, in 200 µl of Tris ethylenediamine tetraacetic acid (EDTA) buffer (10 mM Tris-HCl, pH 8.0 and 0.1 mM EDTA) following previously described protocols (Abdelwhab et al. 2011). Viral RNA was extracted from the FTA cards using the NucleoMag Pathogen kit (Macherey-Nagel, Germany) with an automatic KingFisher Flex Purification System (Thermo Fisher, Waltham, MA; catalog number: 5400630). Samples were tested for AIV-specific RNA using a SYBER Green real-time RT-PCR assay targeting the AIV matrix gene (Fouchier et al. 2000). AIV samples with quantification cycle (Cq) below thirty-four were considered positive and were subsequently tested for the H9 subtype by RT-qPCR (Monne et al. 2008).

The partial hemmaglutinin (*HA*) and neuraminidase (*NA)* gene segments of positive samples were sequenced using Sanger sequencing techniques to select representative strains for full-genome sequencing (data not shown). Selected samples were then subjected to full-genome sequencing using the Illumina MiSeq System (Illumina, San Diego, CA, USA) following previously described protocols (Barman et al. 2019). Seven sequences were generated and manually curated using BioEdit v7.2 (Hall, Biosciences, and Carlsbad 2011).

### Molecular characterization of H9N2 viruses in Mali

To gain insights into the potential determinants of AIV transmission to mammalian species and identify key molecular markers associated with increased virulence, an analysis of the deduced amino acid sequences of the Malian H9N2 viruses was conducted. Specifically, the HA receptor–binding site (RBS) was examined using the H3 numbering system (Burke, Smith, and Digard 2014) to assess its binding affinity to mammalian-type receptors (Wan and Perez 2007; Li et al. 2014). Furthermore, the numbers and locations of N-linked glycosylation sites (Asn-Xaa-Ser/Thr) were determined in the HA segment using the online program NetNGlyc 1.0 (https://services.healthtech.dtu.dk/services/NetNGlyc-1.0/). These glycosylation sites have been associated with AIV antigenic escape from the host’s immune responses (Schulze 1997). We also examined the other gene segments of the H9N2 viruses from Mali to identify amino acid mutations described in experimental studies as associated with increased polymerase activity, enhanced replication in mammalian cells, and increased virulence (PB2-D253N, PB2-I292V, PB2- A588V, PB2- E627K, PB2-D710N, PB1-M185I, PB1-K577E, PA-N291S, PA-K356R, NP-E434K) and antiviral resistance (M2-S31N).

### Continuous phylogeographic analysis of H9N2 in Northern and Western Africa

To confirm whether H9N2 sequences from Western and Northern Africa form a unique clade, we first inferred a maximum likelihood tree (Supplementary Figure S1) from all H9N2 sequences of the HA gene segment available on the Global Initiative on Sharing All Influenza Data database with FastTree v2.1 using the general time-reversible + CAT substitution model (Price, Dehal, and Arkin 2009). H9N2 sequences of the HA gene segment for Northern and Western Africa (Supplementary Table S2), covering the period from 1 January 2011 to 7 February 2022 (*n* = 148) were then merged with the newly generated Malian sequences (*n* = 7). The alignment of the sequences was performed using MAFFT v7.49 (Katoh and Standley 2013) and checked using AliView v1.28 (Larsson 2014). Based on this alignment, a maximum likelihood phylogenetic analysis was first performed using the program RAXML-NG (Kozlov et al. 2019) with 1,000 bootstrap replicates to assess branch support (Supplementary Figure S1). The temporal signal was evaluated using Tempest v1.5.3 (Rambaut et al. 2016). The linear regression of the root-to-tip genetic distance against sampling time showed a strong temporal signal (*R*^2^ = 0.78) (Supplementary Figure S2). This preliminary phylogenetic inference aimed to identify phylogenetic clusters of identical sequences sharing the same geographic coordinates and sampling date. Considering that retaining more than one sequence per cluster would not contribute significant information to subsequent phylogeographic analyses, we subsampled the original alignment to randomly select only one sequence per phylogenetic cluster. As a result, the final dataset of seventy-eight sequences was derived from nine countries in Northern and Western Africa (Algeria, Benin, Ghana, Mali, Morocco, Nigeria, Senegal, Togo, and Tunisia), with sampling dates ranging from 2 April 2012 to 7 February 2022 (Supplementary Table S1). To avoid duplication of sampling coordinates in this final dataset, we randomly sampled data within a circular buffer zone of 0.25, excluding the sea areas if they fell within the buffer (Dellicour et al. 2020).

The continuous phylogeographic analysis was performed using Bayesian evolutionary analysis by sampling trees v1.10.4 (Drummond and Rambaut 2007) along with the BEAGLE 3 library to improve computational performance (Ayres et al. 2019). The substitution process was modeled using the HKY + Γ4 parameterization (Shapiro, Rambaut, and Drummond 2006), the branch-specific evolutionary rates were modeled using a relaxed molecular clock with a lognormal distribution (Drummond et al. 2006), and a skygrid coalescent model was specified as tree topology prior (Hill and Baele 2019). We used the relaxed random walk (RRW) diffusion model (Lemey et al. 2010; Pybus et al. 2012) to perform the continuous phylogeographic reconstruction, with the among-branch heterogeneity in diffusion velocity modeled with a gamma distribution. The RRW model does not allow different sequences to be associated with identical geographical coordinates. Therefore, a spatial buffer zone of 25 km was applied to the locations of the sequences to prevent improper posteriors under the RRW model, excluding sea areas falling within the buffer (Dellicour et al. 2020). We ran and combined three independent analyses for one billion generations, sampling every 100,000 generations. Convergence and mixing properties were again assessed using Tracer v1.7.1 (Rambaut et al. 2018), ensuring that all continuous parameters were associated with an effective sample size value of >200. After having discarded 10 per cent of sampled posterior trees as burn-in, we obtained and annotated the maximum clade credibility (MCC) tree using TreeAnnotator 1.10.4 (Suchard et al. 2018). We identified phylogenetic trees that exhibit multiple long-distance dispersal events by examining the latitudes of branches in the trees. We used the latitude of 21.3 as a cut-off, which was in a wide swath of geography between our Northern and Western Africa samples. Trees with more than one branch starting above the cut-off and ending below it are considered to have multiple long-distance dispersal events. We used functions available in the R package ‘seraphim’ (Dellicour et al. 2016, 2017) to extract spatio-temporal information embedded within posterior trees and visualize the continuous phylogeographic reconstructions. We also used the ‘spreadStatistics’ function of the R package ‘seraphim’ to estimate the spatial wavefront distance from the epidemic origin, the weighted lineage dispersal velocity over time, and the weighted diffusion coefficient (Trovão et al. 2015).

Given the sensitivity of phylogeographic analyses to heterogeneous sampling efforts (Kalkauskas et al. 2021), we investigated the potential impact of different sampling strategies on the estimation of clock rate and time to the most common recent ancestor parameters, as well as on the topology of the MCC tree. Two sampling schemes were employed: (1) random sampling for equivalent representation: sequences were randomly selected to ensure an equivalent number of sequences from both Northern and Western Africa to achieve balanced representation from these two regions (*n* = 32 sequences in each region to allow an equal number of sequences per region while using most of the available sequences) and (2) random sampling for reduced biased sampling: sequences were randomly sampled to reduce the number of sequences from Morocco where high sampling was observed compared to other countries to mitigate any potential bias arising from uneven sampling effort across different countries (*n* = 13 sequences in Morocco to ensure that this country did not have the highest number of sequences). We ran three independent analyses for each sampling scheme to explore variability.

## Results

### Identification and whole-genome sequencing of Malian H9N2 viruses

We screened 519 oropharyngeal samples collected from LBMs by RT-qPCR. Eighty-four samples (16 per cent) tested positive for AIV specifically for the H9 subtype with Cq values ranging from 20.7 to 35.4. Seven positive samples (Table 1) were selected for whole-genome sequencing and fully sequenced. The nucleotide sequence identity among the Malian H9N2 viruses ranged from 95.8 to 100 per cent in the eight gene segments. Sequences generated in this study were submitted to GenBank (accession numbers: OR133241–OR133296).

**Table 1.** H9N2 viruses characterized in this study.

Sample ID	Collection date	Location	Accession number
A/chicken/Mali/22-A-01-113/2022	8 January 2022	Ségou	OR133289–96
A/chicken/Mali/22-A-02-123/2022	7 February 2022	Bamako	OR133241–48
A/chicken/Mali/22-A-02-128/2022	7 February 2022	Bamako	OR133249–56
A/chicken/Mali/22-A-02-139/2022	7 February 2022	Bamako	OR133257–64
A/chicken/Mali/22-A-02-143/2022	7 February 2022	Bamako	OR133265–72
A/chicken/Mali/22-A-02-152/2022	7 February 2022	Bamako	OR133273–80
A/chicken/Mali/22-A-02-158/2022	7 February 2022	Bamako	OR133281–88

### Molecular characterization of Malian H9N2 viruses

A detailed analysis of the deduced amino acid sequences of Malian H9N2 viruses identified certain molecular markers associated with mammalian adaptation, enhanced replication, and antiviral resistance in the HA and M proteins (Table 2). In particular, all Malian H9N2 viruses carry the Q226L and I155T mutations (H3 numbering) in the RBS of the HA protein. These mutations are involved in the binding of AIVs to mammalian receptors and enhanced replication in mammalian cells and ferrets (Table 2). On the contrary, T190V substitution, associated with enhanced binding affinity for the human-type sialic acid receptor and replication, was absent in the H9N2 virus of this study. The HA protein of five Malian H9N2 viruses had the RSSR/GLF amino acid motif at the cleavage site, which is characteristic of LPAIV of H9 G1 lineage (Banks et al. 2000). Conversely, two Malian H9N2 viruses harbor the RSNR/GLF motif due to the substitution of S329N (H3 numbering). Although the RSNR motif had previously been reported in various studies on avian H9N2 viruses in Asia (Perk et al. 2006; Tosh et al. 2008; Butt et al. 2010), to our knowledge, this is the first report of this motif in H9N2 viruses from Africa. Regarding the other proteins of H9N2 viruses, the well-known mammalian adaptation markers, PB2-E627K and D701N, were not found in any of the H9N2 viruses from Mali (Table 2). In addition, PB2-D253N, PB2-I292V, PB2- A588V, PB1-K577E, PA-K356R, and NP-E434K mutations associated with either increased polymerase activity in mammalian cells or increased virulence were not present in H9N2 virus from Mali. In contrast, S31N mutation in the M2 protein associated with amantadine resistance and M185I in PB2 and N291S in PA associated with increased pathogenicity in mice were observed in the seven H9N2 viruses from Mali (Table 2). Based on the results predicted using NetNGlyc 1.0 software, all the Malian H9N2 viruses had seven N-linked glycosylation sites in the HA protein at positions (29, 141, 218, 298, 305, 492, and 551).

**Table 2.** Amino acid substitutions associated with host-shift and virulence in the Malian H9N2 viruses.

Gene	Mutation	A-01-113	A-02-123	A-02-128	A-02-139	A-02-143	A-02-152	A-02-158	Functions
HA	**I155T** (145)^a^	**T**	**T**	**T**	**T**	**T**	**T**	**T**	Binding of AIV to mammalian receptors (Li et al. 2014)
	T190V (180)	A	A	A	A	A	A	A	Enhanced binding affinity to mammalian cells and replication in mammalian cells (Teng et al. 2016)
	**Q226L** (216)	**L**	**L**	**L**	**L**	**L**	**L**	**L**	Increased virus binding to α2–6, enhanced replication in mammalian cells and ferrets, enhanced transmission in ferrets (Wan and Perez 2007; Wan et al. 2008; Peacock et al. 2021)
	HA1/HA2 cleavage site	R-S-N-R	R-S-R-R	R-S-N-R	R-S-R-R	R-S-R-R	R-S-R-R	R-S-R-R	
PB2	**M185I**	**I**	**I**	**I**	**I**	**I**	**I**	**I**	Enhanced pathogenicity in mice (Zhang et al. 2011)
	D253N	D	D	D	D	D	D	D	Increased polymerase activity in mammalian cell lines (Zhang et al. 2018)
	I292V	I	I	I	I	I	I	I	Increased polymerase activity in mammalian cell lines and increased virulence in mice (Gao et al. 2019)
	A588V	A	A	A	A	A	A	A	Increased polymerase activity and replication in mammalian, increased virulence in mice (Xiao et al. 2016)
	E627K	E	E	E	E	E	E	E	Increased virulence and transmissibility in mammals, increased polymerase activity (Sang et al. 2015a; Sediri et al. 2016)
	D701N	D	D	D	D	D	D	D	Increased transmissibility in mammals (Sediri et al. 2016)
PB1	K577E	K	K	K	K	K	K	K	Increased polymerase activity and virulence in mice (Kamiki et al. 2018)
PA	**N291S**	**S**	**S**	**S**	**S**	**S**	**S**	**S**	Enhanced pathogenicity in mice (Zhang et al. 2011)
	K356R	K	K	K	K	K	K	K	Increased polymerase activity and enhanced replication in mammalian cell lines, increased virulence in mice (Xu et al. 2016)
NP	E434K	E	E	E	E	E	E	E	Increased polymerase activity in mammalian cell lines (Sang et al. 2015b)
M2	**S31N**	**N**	**N**	**N**	**N**	**N**	**N**	**N**	Amantadine resistance (Ilyushina, Govorkova, and Webster 2005)

Mutations associated with mammalian adaptation or virulence are highlighted in bold.

a Mature H3 HA numbering (mature H9 HA numbering).

### Phylogenetic and phylogeographic analyses

**Figure 1.**
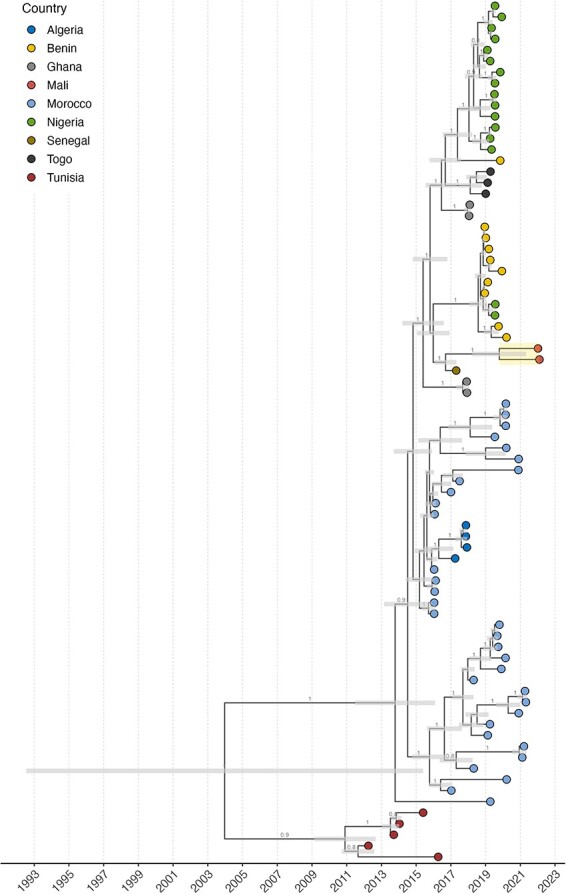
MCC tree estimated from H9N2 genome sequences collected from nine countries in Northern and Western Africa (Algeria, Benin, Ghana, Mali, Morocco, Nigeria, Senegal, Togo, and Tunisia) from 2 April 2012 to 7 February 2022. Tip node colors indicate the country of sampling. Bars at internal nodes represent the 95 per cent highest posterior density intervals of the node date. Numbers at internal branches show the clade posterior probabilities above 0.75. The two tip nodes highlighted show the Malian sequences.

The phylogenetic analysis of the *HA* gene segment (Fig. 1) showed that the Malian H9N2 viruses belong to the H9N2 G1 lineage and were closely related to viruses isolated in Western Africa between 2017 and 2019. The time to tMRCA of the *HA* gene segment for Malian H9N2 viruses was estimated around 2019 with 95 per cent Highest Posterior Distribution (HPD) between 2018 and 2021. The tree topology indicated that the Malian strains had evolved into two genetically distinct clusters within the country, comprising five and two sequences, respectively, out of the seven newly generated sequences (Supplementary Figure S1). It also shows that sequences from Morocco were those most closely related to the West African sequences. The tMRCA of the H9N2 virus in Northern and Western Africa was estimated to have occurred in December 2003 (95 per cent HPD: May 1989 to April 2012), and the median evolutionary rate was estimated to be 3.8 × 10^−3^ substitutions per site per year (95 per cent highest posterior density HPD: 2.7 × 10^−3^ to 35.2 × 10^−3^).

**Figure 2.**
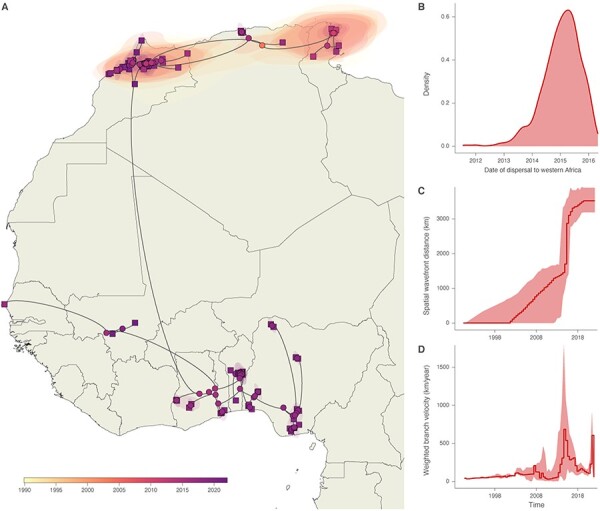
Continuous phylogeographic reconstruction of the dispersal history of H9N2 lineages in Northern and Western Africa. We here map the MCC tree and 80 per cent highest posterior density regions (A) reflecting the uncertainty related to the Bayesian phylogeographic inference. Nodes shaped as circles and squares indicate internal and tip nodes, respectively, and are colored according to their time of occurrence. This figure also reports dispersal statistics at the bottom: the date of the dispersal event to Western Africa (B), the evolution of the spatial wavefront distance from the origin of the epidemic (C), and the evolution of the weighted lineage dispersal velocity through time (D).

Phylogeographic reconstructions revealed a single significant long-distance lineage dispersal event that consistently appeared in all posterior trees (Fig. 2). The long-distance dispersal event was followed by the spread of H9N2 lineages across various countries in Western Africa (Fig. 2). The estimations of weighted lineage dispersal velocity revealed temporal variations in the rate of spread. From 2003 to 2013, the median weighted lineage dispersal velocity was approximately 92 km/year (95 per cent HPD 30–208). However, from 2014 onwards, the dispersal velocity exhibited an increased trend over time, with a notable increase up to 685 km/year (95 per cent HPD 7–1,789) observed around 2015 (Fig. 2). This increase corresponds to the expansion phase of the epidemic from Northern to Western Africa. Subsequently, between 2017 and 2020, the dispersal velocity decreased to approximately 150 km/year (95 per cent HPD 107–229), followed by a second notable increase observed in 2021, reaching up to 607 km/year (95 per cent HPD 200–905). The observed temporal changes in weighted lineage dispersal velocity were further supported by our analysis of the spatial wavefront distance from the epidemic origin. This analysis showed an approximate 1,000 km increase in the distance covered by lineages around 2015 (Fig. 2). Additionally, the median weighted diffusion coefficient was 35,084 km^2^/year (95 per cent HPD 23,962–49,492).

The sensitivity analysis revealed that the choice of sampling schemes had a minimal impact on our conclusions related to the spatio-temporal origin of the H9N2 lineages spread. The single significant long-distance lineage dispersal event originating from Morocco was observed in all posterior trees in the reduced sampling scheme (Supplementary Figure S3) and 98.5 per cent (985 out of 1,000) of posterior trees in the equivalent sampling scheme (Supplementary Figure S4). The median rate of evolutionary change ranged from 3.8 × 10^−3^ to 4.2 × 10^−3^ substitutions per site and per year, with overlapping 95 per cent HPD intervals across all sampling schemes (Supplementary Figure S4). Only the estimated median tMRCA varied slightly across the different sampling schemes due to the reduced number of sequences, ranging from December 2003 to December 2005, which represents a relatively short time scale related to the studied period.

## Discussion

The G1 lineage has been known to be circulating in Africa since 2016 and has spread to different countries within the continent (El Houadfi et al. 2016; Zecchin et al. 2017; Awuni et al. 2019; Fusade-Boyer et al. 2021; Sulaiman et al. 2021). Previous studies reported the circulation of AIVs among domestic and wild birds in Mali, but they have mainly focused on HPAIV strains and did not provide sufficient information on the genetic and molecular characteristics of the circulating viruses (Molia et al. 2010, 2017; Cappelle et al. 2012).

The positivity rate of AIVs among poultry in LBMs (16 per cent) was higher than the AIV prevalence previously reported in backyard poultry (3.6 per cent) in Mali (Molia et al. 2010). However, our result should be interpreted cautiously since the number of samples collected was very limited and was not representative of the whole country. In addition, in our study, genetic characterization was performed on samples from FTA cards, which could reduce the viral load and affect the number of positive samples. The use of FTA cards also made it impossible to culture and isolate the virus, as FTA cards inactivate the virus while preserving the nucleic acids (Abdelwhab et al. 2011). Despite these limitations, FTA cards were essential to overcome the technical difficulties of maintaining the cold chain and storage.

LBMs are considered a potential reservoir for AIVs that can play a major role in their amplification and dissemination among poultry (Molia et al. 2016; Sulaiman et al. 2021). Additionally, Malian LBMs were characterized by the main risk factors associated with the presence of AIVs such as poor biosecurity and hygienic practices and large catchment areas of backyard poultry (Molia et al. 2016). Thus, it is possible that after being introduced in the LBMs, AIVs were amplified and more easily spread among birds.

Analysis of the amino acid sequences of the HA protein of the five out of seven Malian H9N2 viruses indicated the presence of the RSSR/GLF motif at the HA cleavage site, which is identical to those found in the LPAI H9N2 viruses circulating in Western Africa and elsewhere. However, two Malian H9N2 viruses had a different pattern (RSNR/GLF) at their cleavage site. To the best of our knowledge, this pattern was described only in H9N2 viruses isolated in humans and poultry in Israel and India (Perk et al. 2006; Tosh et al. 2008). The significance of this mutation on viral fitness and pathogenicity is not fully understood, and further study is needed. Nevertheless, as the cleavage site is an indicator of pathogenicity, these findings may indicate a change in the degree of virulence of these two viruses (Zhang et al. 2021).

All the Malian H9N2 viruses possess amino acid residues in the HA RBS that were associated with an increased binding affinity of AIVs to human-type (α-2,6-linked sialic acids) receptors such as L-226 and T155 (Wan and Perez 2007; Li et al. 2014; Peacock et al. 2021), indicating the potential for these viruses to infect humans as reported in previous studies (Saito et al. 2001; Jallow et al. 2020). These mutations were observed in recent H9N2 viruses belonging to the G1-lineage isolated in many countries in Africa (Awuni et al. 2019; Kariithi et al. 2020; Sulaiman et al. 2021; Atim et al. 2022) confirming their spread within the region. Of note, most of the H9N2 viruses of the G1 lineage detected after 2,000 and almost all the H9N2 viruses identified in humans and other mammalian species such as horse, dog, and mink, harbor leucine at position 226 confirming preferential binding to mammalian receptors (Chrzastek et al. 2018; Pusch and Suarez 2018). Mutations associated with increased pathogenicity in mice were observed in PB2 (M185I) and PA (N291S), confirming the ability of these H9N2 viruses to infect mammalian species (Zhang et al. 2011).

All the H9N2 viruses from Mali have seven N-linked glycosylation sites (positions 21, 97, 133, 290, 297, 484, and 543, H3 numbering), which were identical to those found in H9N2 viruses isolated in Northern Africa (Barberis et al. 2020; Larbi et al. 2022). The additional glycosylation sites reported in some H9N2 strains isolated in Western Africa (Zecchin et al. 2017; Awuni et al. 2019; Fusade-Boyer et al. 2021) were not observed in the Malian H9N2 viruses. Compared to G1-like prototypes (A/quail/Hong Kong/G1/97), the Malian H9N2 viruses lost two glycosylation sites at positions 198 and 210. Glycosylation plays an important role in viral biology, regulating the virulence and receptor-binding specificity of AIVs (Schulze 1997; Peng et al. 2019). Variations in the number or position of glycosylation sites may affect the biology of AIVs and could allow these viruses to escape host antibody recognition (Peng et al. 2019).

This study also explores the spatio-temporal spread of H9N2 lineages in Northern and Western Africa. We performed continuous phylogeographic reconstructions based on the analysis of H9N2 sequences of the *HA* gene segment, including recent sequences generated from Mali. The tMRCA of the H9N2 virus in Northern and Western Africa was estimated around December 2003 (95 per cent HPD: May 1989 to April 2012), while the first detection in these regions occurred in 2015/2016. This time difference may be explained by the low pathogenicity of the H9N2 virus in poultry and wild birds and its mostly asymptomatic circulation, making it difficult to detect through routine surveillance (Peacock et al. 2019; Fusade-Boyer et al. 2021).

In addition, active surveillance for H9N2 viruses is limited and the virus is usually detected only when it is associated with mortality in poultry (El Houadfi et al. 2016; Jeevan et al. 2019).

The Malian H9N2 viruses closely clustered with H9N2 viruses circulating in Western Africa (Zecchin et al. 2017; Awuni et al. 2019; Fusade-Boyer et al. 2021) and Northern Africa (El Houadfi et al. 2016; El Mellouli et al. 2022b). Most of the borders in Western Africa are known to be porous with illegal movements of animals without any border controls (Apolloni et al. 2019). Therefore, it can be assumed that these viruses most likely originated from the region and might have been introduced in Mali from the neighboring countries by cross-border poultry movement and trade. Our findings also highlight the occurrence of a single long-dispersal event around 2015, during which the virus spread from Morocco to Western Africa at a velocity of approximately 685 km/year. This dispersal event likely originated from Morocco, serving as the primary source for the subsequent expansion phase of H9N2 lineages from Northern to Western Africa. While the involvement of long-distance migratory wild birds during autumn migration could be a potential contributing factor to the dissemination of H9N2 to Western Africa, the likelihood of such virus dispersal over very long distances by wildfowl is low and estimates of wild bird migration velocities are higher (Gaidet et al. 2010; Lemke et al. 2013; Trierweiler et al. 2014). In addition, during active surveillance in wild birds in Morocco between 2016 and 2019, AIVs were found to be circulating at a very low prevalence (1.83 per cent) and no H9N2 virus was detected (El Mellouli et al. 2022a).

Given that H9N2 viruses become well adapted to poultry, it is more likely that the dispersal event was influenced by anthropogenic factors, specifically the movement of live poultry, facilitating the transmission of H9N2 to Western Africa (Peacock et al. 2019). Notably, several countries in Western Africa import hatching eggs and day-old chicks from Morocco, and there has been a significant increase in import quantities between 2013 and 2015 (Supplementary Figure S6) (Hassan et al. 2017; FAO 2023). It is therefore likely that such trade activities have contributed to the introduction of H9N2 viruses into the region. This result confirms previous studies suggesting the primary role played by Morocco in the spread of H9N2 viruses in Africa (Zecchin et al. 2017; El Mellouli et al. 2022b). However, the lack of comprehensive poultry trade data limits our ability to conduct a robust investigation into the influence of live poultry trade on lineage dispersal. Future studies incorporating more detailed and comprehensive trade data would provide valuable insights into the dynamics of H9N2 virus dissemination in the region.

Our results should be interpreted in light of some limitations. In particular, the overall size of the genome data was limited, resulting in omitting transmission events and increased uncertainty in our analyses. Notably, some countries in the affected region were unsampled, potentially missing important transmission dynamics. For example, Ivory Coast, which is closely connected to the sampled countries according to poultry trade data (Supplementary Figure S5), lacks representation in our samples. Additionally, we lack sampling from Niger and Mauritania, which shared borders with affected countries in both Western and Northern Africa. This implies the possibility of unobserved dispersal events between intermediate countries. This limitation also applies to the identified long-distance dispersal event from Morocco to Western Africa, although the crossing region is a deserted area with a limited poultry population along the way (Robinson et al. 2014). The estimation of velocity during this period including the long-distance dispersal event is also subject to heightened uncertainty, primarily attributed to the limited number of sequences around this event, adding complexity to the modeling process. Furthermore, the lack of sequences before 2012 introduced additional uncertainties before that date. To address these limitations, we advocate for an intensified genomic surveillance of H9N2 in the region. This would facilitate more thorough phylogeographic reconstructions and contribute to a more comprehensive understanding of the virus’s spread both in poultry and potentially in wild bird populations.

The H9N2 virus has become endemic in several countries around the world and occasionally causes outbreaks in poultry, resulting in huge economic losses to the poultry industry. In Western Africa, the surveillance of H9N2 viruses is very limited, making it difficult to obtain reliable data on the genetic characteristics and transmission patterns of this virus in the region. In addition, the current circulation of HPAI H5Nx of clade 2.3.4.4b could contribute to the emergence of reassortant strains, as recently demonstrated in Burkina Faso (Ouoba et al. 2022), posing a threat to human and animal health. Therefore, long-term surveillance of the H9N2 virus in poultry populations in Western Africa, particularly in LBMs, is crucial for an improved understanding of viral evolution and effective management against potential zoonotic AIV strain emergence.

## Supplementary Material

veae011_Supp

## Data Availability

All Malian H9N2 genome sequences are available on GenBank under accession numbers: OR133241–OR133296 (https://www.ncbi.nlm.nih.gov/nuccore/). H9N2 genome sequences used in this study are available on the GISAID database (http://www.gisaid.org). The BEAST 1 XML file used to perform the phylogeographic analysis and the R scripts are available from https://github.com/ClaireGuinat/h9n2_continous_phylo.git.
