# Supplementary material for: Genetic insights of H9N2 avian influenza viruses circulating in Mali and phylogeographic patterns in Northern and Western Africa
Source: Virus Evol. 2024 Feb 19;10(1):veae011. doi: 10.1093/ve/veae011 (PMC10908551; doi:10.1093/ve/veae011)
Supplement: veae011_Supp [file veae011_supp.zip › suppl_data/Supplementary information_Rev_VEVOLU-2023-164-R2.docx]

**Supplementary information**

**Table S1**. Descriptive summary of the final dataset of 78 sequences derived from 9 countries in Northern and Western Africa used for phylogeographic analysis

| **Country** | **Number** | **Minimum date** | **Maximum date** |
| --- | --- | --- | --- |
| Algeria | 4 | 01-04-2017 | 15-12-2017 |
| Benin | 10 | 15-12-2018 | 17-03-2020 |
| Ghana | 4 | 29-11-2017 | 02-02-2018 |
| Mali | 2 | 08-01-2022 | 07-02-2022 |
| Morocco | 33 | 15-01-2016 | 28-04-2021 |
| Nigeria | 16 | 08-02-2019 | 07-12-2019 |
| Senegal | 1 | 23-04-2017 | 23-04-2017 |
| Togo | 3 | 04-01-2019 | 15-04-2019 |
| Tunisia | 5 | 02-04-2012 | 10-04-2016 |


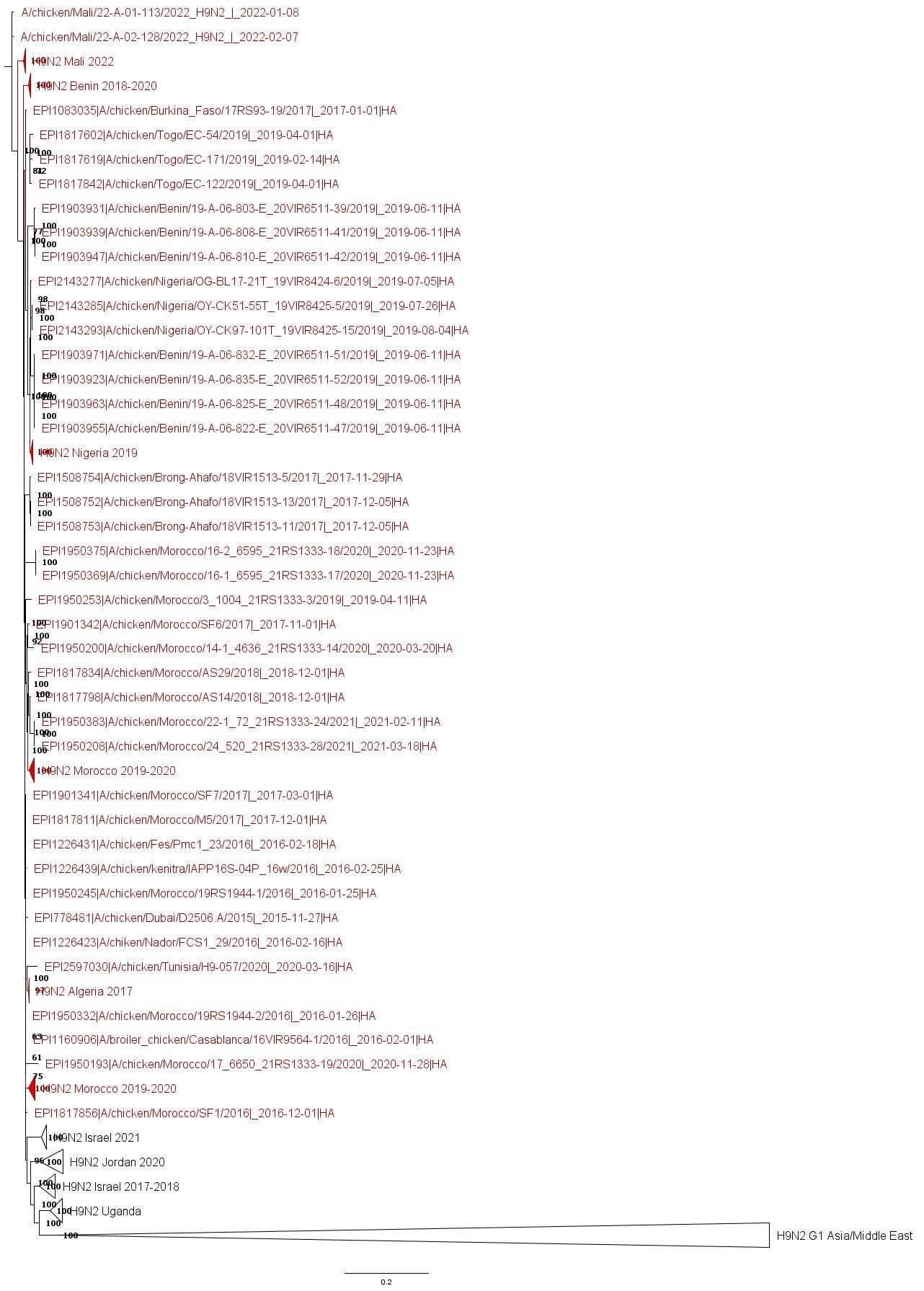


Figure S1. Plot of the maximum likelihood phylogenetic tree for all the H9N2 sequences from GISAID. Sequences for Western and Northern Africa are depicted in red. Bootstrap values ≥ 60% are shown near the nodes. Scale bar indicates the average number of nucleotide substitutions per site.


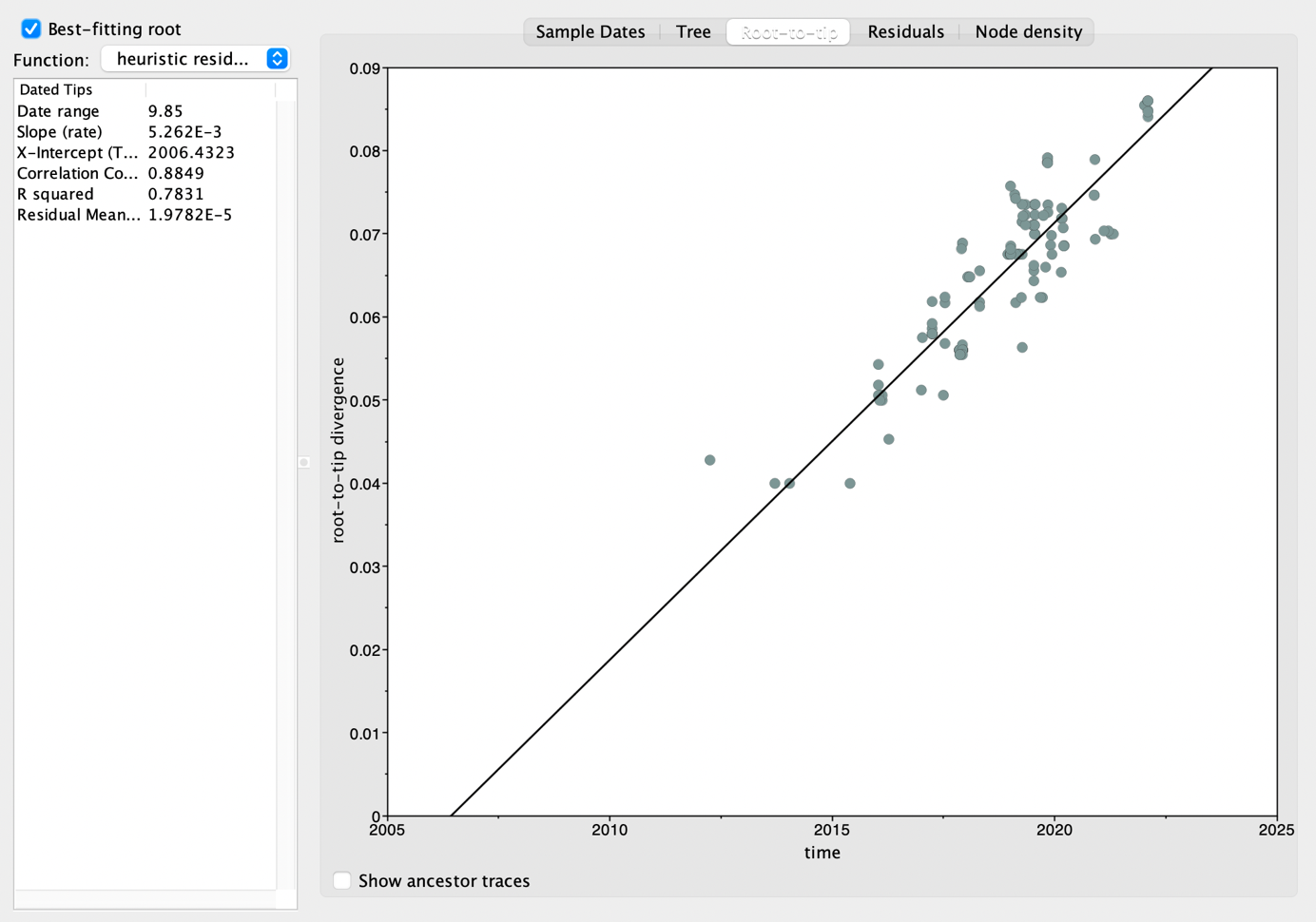


**Figure S2.** Plot of the root-to-tip genetic distance against sampling time for the dataset of 155 sequences


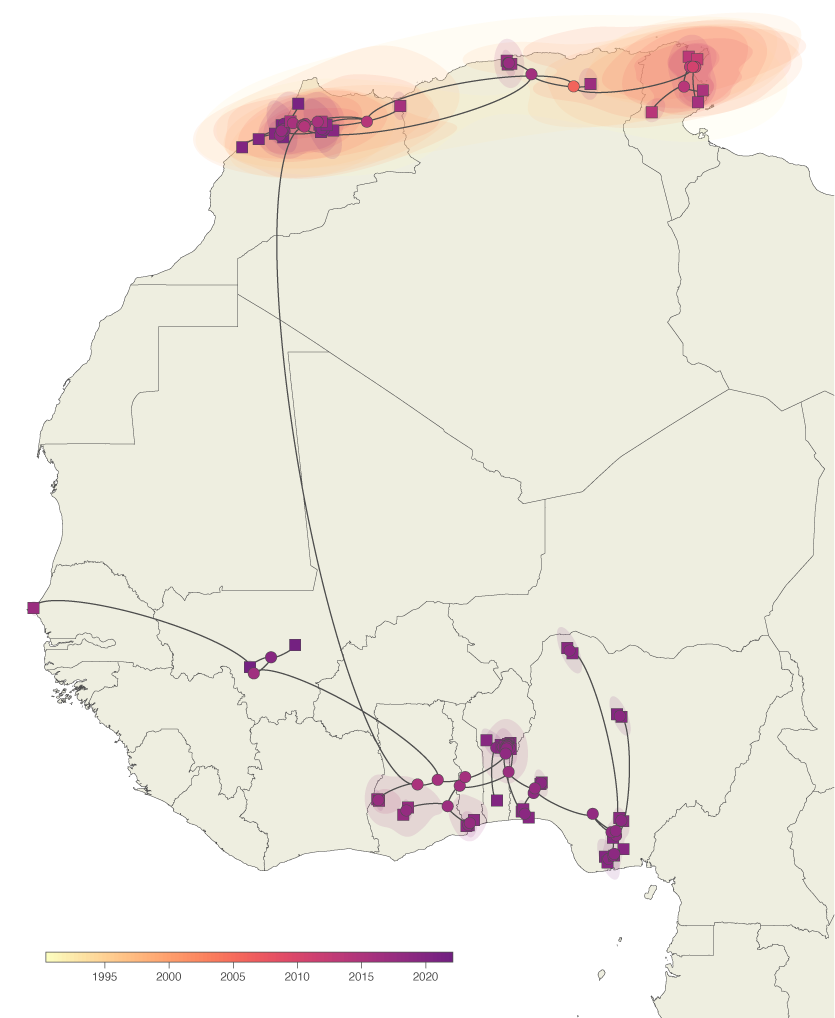


**Figure S3**. Continuous phylogeographic reconstruction of the dispersal history of H9N2 lineages in Northern and Western Africa, here based on random sampling for reduced biased sampling. We here map the maximum clade credibility (MCC) tree and 80% highest posterior density regions reflecting the uncertainty related to the Bayesian phylogeographic inference. Nodes shaped as circles and squares indicate internal and tip nodes, respectively, and are colored according to their time of occurrence.


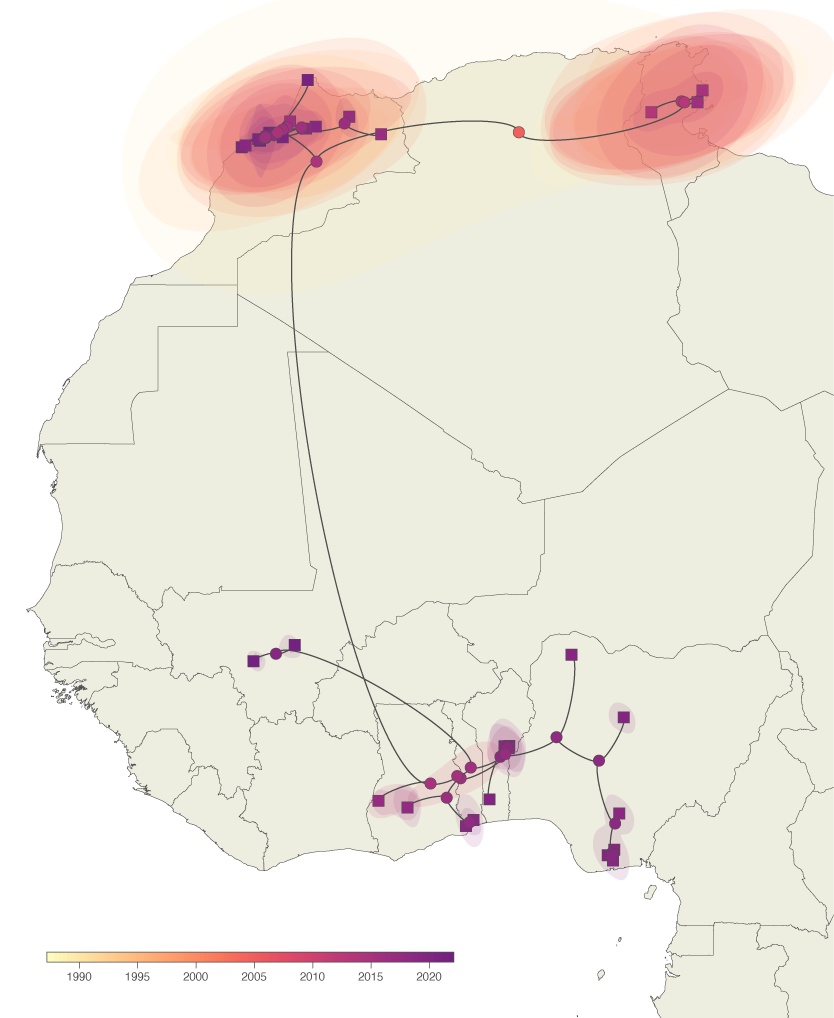


**Figure S4.** Continuous phylogeographic reconstruction of the dispersal history of H9N2 lineages in Northern and Western Africa, here based on random sampling for equivalent representation. We here map the maximum clade credibility (MCC) tree and 80% highest posterior density regions reflecting the uncertainty related to the Bayesian phylogeographic inference. Nodes shaped as circles and squares indicate internal and tip nodes, respectively, and are colored according to their time of occurrence.


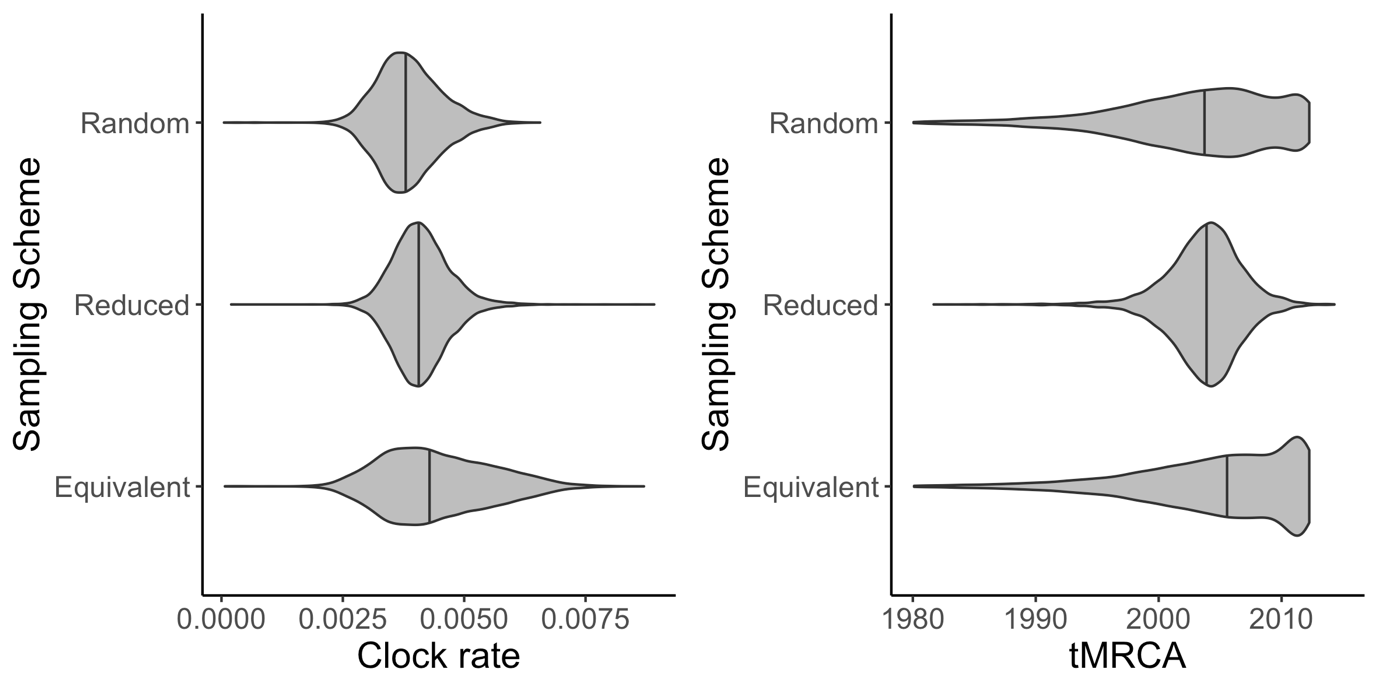


**Figure S5.** Clock rate and time to the most recent common ancestor (tMRCA) estimated by different sampling schemes: random sampling, random sampling for equivalent representation, and random sampling for reduced biased sampling. The central line represents the posterior median.


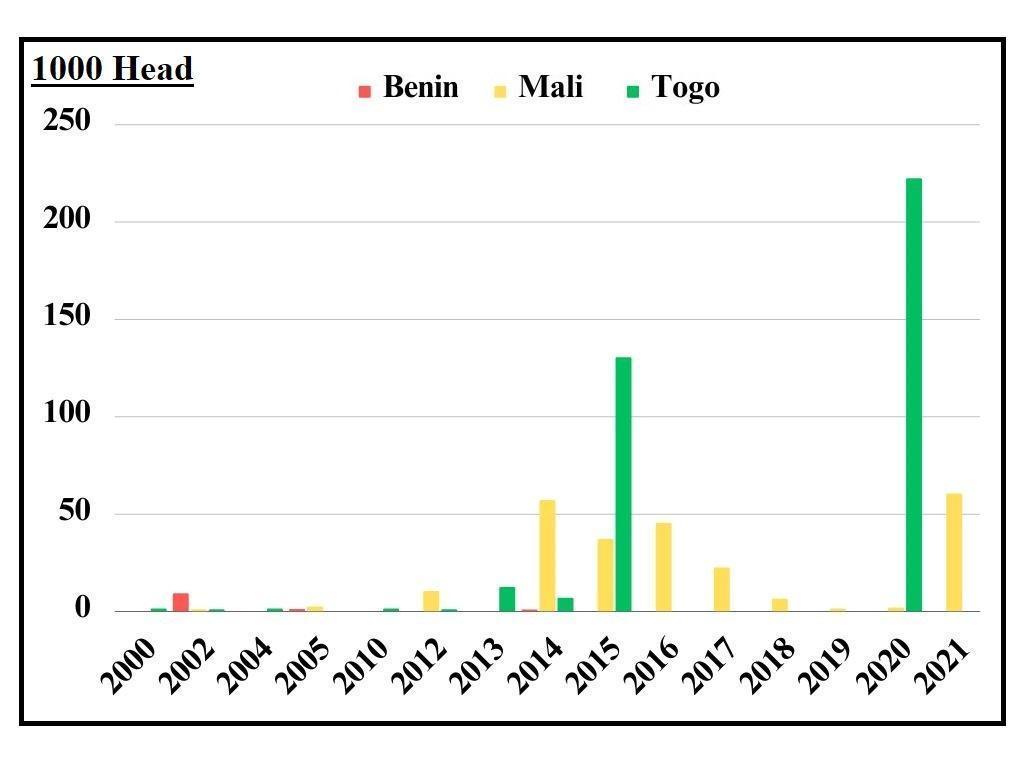


**Figure S6.** Import of hatching eggs and day-old chicks from Morocco. Data from (FAO, 2023)
